# Tic-Related Obsessive–Compulsive and Eating Disorders in Dandy–Walker Variant: A Case Report and Systematic Reappraisal of Psychiatric Profiles

**DOI:** 10.3390/brainsci14040362

**Published:** 2024-04-06

**Authors:** Riccardo Bortoletto, Anna Candolo, Alessandra Nicotra, Luana Saetti, Laura Perini, Matteo Balestrieri, Marco Colizzi, Carla Comacchio

**Affiliations:** 1 Unit of Psychiatry, Department of Medicine (DMED), University of Udine, 33100 Udine, Italy; bortoletto.riccardo@spes.uniud.it (R.B.); candoloanna@gmail.com (A.C.); nicotra.alessandra@asufc.sanita.fvg.it (A.N.); luana.saetti@asufc.sanita.fvg.it (L.S.); laura-perini@libero.it (L.P.); matteo.balestrieri@uniud.it (M.B.); carla.comacchio@asufc.sanita.fvg.it (C.C.); 2 Department of Psychosis Studies, Institute of Psychiatry, Psychology and Neuroscience, King’s College London, London SE5 8AF, UK

**Keywords:** neurodevelopment, intellectual disability, cerebellum, mega cisterna magna, Blake pouch cyst, schizophrenia, antipsychotics, mood stabilizers, antidepressants

## Abstract

Dandy–Walker complex (DWC) consists of a continuum of brain malformations involving the posterior fossa, often leading to psychiatric manifestations during adulthood. We discussed the case of a young woman with Dandy–Walker variant (DWV) and a comorbid complex neuropsychiatric presentation, who was diagnosed with an eating disorder, obsessive–compulsive disorder, and a tic disorder. Afterwards, we conducted a Preferred Reporting Items for Systematic Reviews and Meta-Analyses (PRISMA) 2020-compliant systematic review reappraising all evidence of psychiatric outcomes in adults with DWC. Overall, 34 studies were eligible for data extraction, comprising 36 patients. Psychiatric profiles were more common among young adult males, with DWC lesions, especially DWV subtype, being often discovered incidentally after admission to mental health inpatient facilities. Most patients were diagnosed with psychosis and bipolar disorder, often comorbid with cognitive impairment. Psychotropic polypharmacy was frequently prescribed, generally leading to complete recovery. Evidence from our case report and systematic review indicates the importance of monitoring long-term psychiatric sequelae among adult patients with DWC malformations.

## 1. Introduction

**Figure 1:**
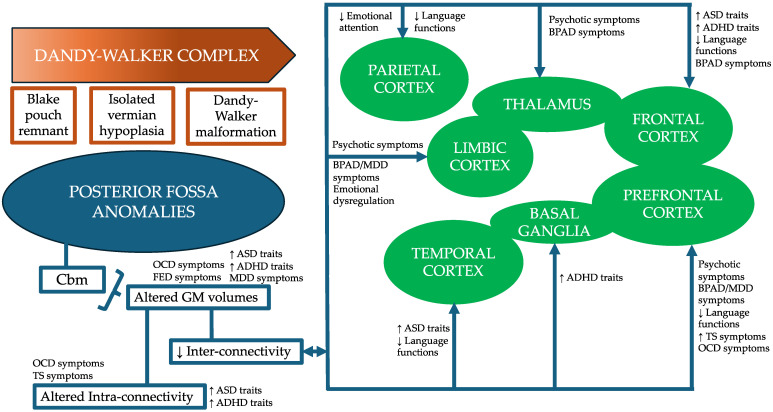
Dandy–Walker complex represents a continuum of posterior fossa anomalies, primarily characterized by abnormal cerebellum development, with implications for several neuropsychiatric disorders. ↑, increase; ↓, decrease; ADHD, Attention-Deficit/Hyperactivity Disorder; ASD, Autism Spectrum Disorder; BPAD, Bipolar Affective Disorder; Cbm, Cerebellum; FED, Feeding and Eating Disorder; GM, Gray Matter; MDD, Major Depressive Disorder; OCD, Obsessive–Compulsive Disorder; TS, Tourette syndrome.

Dandy–Walker complex (DWC) defines a spectrum of congenital multifactorial anomalies of the posterior fossa [1]. According to the modern classification, DWC includes Dandy–Walker malformation (DWM), isolated vermian hypoplasia, and Blake pouch remnant [2,3]. In contrast, the previous nomenclature identified four different clinical entities standing alongside each other in a continuum, comprising DWM, Dandy–Walker variant (DWV), mega cisterna magna (MCM), and posterior fossa arachnoid cyst (PFAC) [4]. To date, this terminology is considered controversial and it is preferable to provide an accurate description of the specific structural abnormalities observed in each patient, with the abnormal development of the cerebellar vermis being considered the standard reference point [5]. The clinical presentation of DWC is heterogeneous and influenced by multiple factors, including the severity of hydrocephalus, intracranial hypertension, cerebellum-related motor and coordination deficits, and associated neurodevelopmental and behavioral comorbidities [6]. Indeed, both cerebellar gray matter volume reduction [7,8,9] and disrupted connections to specific brain areas (e.g., prefrontal, superior temporal, posterior parietal, limbic cortices) [7,10] underpinning social functions [11,12], language [10,11,13], repetitive behaviors [8], cognitive processes [11,13], and affective regulation [12,13], seem to be implicated in several neuropsychiatric conditions, including autism spectrum disorder (ASD), attention-deficit/hyperactivity disorder (ADHD), schizophrenia, and mood disorders (Figure 1).

Increasing evidence indicates that psychiatric manifestations are not uncommon in the context of DWC abnormalities. However, their persistence through late adolescence and adulthood is less clear. The purpose of this work is twofold: (i) to report the case of a 23-year-old woman of Asian ethnicity with DWV, presenting to the Unit of Psychiatry outpatient service at the University Hospital of Udine (Italy) for persisting ruminating thoughts and repetitive behaviors; (ii) to summarize all available data generated by case reports and/or case series investigating psychiatric outcomes in adults with DWC abnormalities by carrying out a systematic literature search for all such data.

## 2. Case Presentation

### 2.1. Medical History

The patient and her mother provided the medical history. She was living with her parents, her twin brother, and her younger sister. The family is of Iraqi origin and moved to Italy when the patient was four years old. Her parents are first cousins. Her family history is positive for mild to moderate intellectual disability in her twin brother and maternal aunt, and severe intellectual disability in her younger sister. Additionally, both her siblings were diagnosed with Chudley–McCullough Syndrome, an autosomic recessive condition sharing some common neurobiological and anatomical features with DWC (e.g., hydrocephalus, cerebellar dysgenesis). Their condition was investigated through Next Generation Sequencing (NGS) analysis, showing G Protein Signaling Modulator (GPSM)-2 gene intron 9 variant c.1062+3A>C at the homozygous state. As displayed through segregation analysis, the patient and her father presented the same variant at the homozygous state, thus ruling out its pathogenic significance. Also, no causative mutations were detected through subsequent clinical exomes in either of the patient’s siblings. She was born preterm (after seven months of gestation) out of her parents’ first pregnancy, with the need for prolonged hospitalization after birth. She presented delayed expressive and receptive language development. Allegedly, all other developmental milestones were reached on time. The occurrence of motor or vocal tics during childhood was denied. At the age of seven, she underwent a brain magnetic resonance imaging (MRI) scan, which showed DWV, comprising enlargement of the posterior pericerebellar cerebrospinal fluid (CSF) spaces with associated widening of the foramen of Magendie and inferior vermis hypoplasia, as well as enlargement of the fourth ventricle and lower sectors of Sylvius’ aqueduct. No failures, difficulties, or low grades were reported throughout the school years. Conversely, premorbid social adjustment was described as subnormal: the patient’s social interactions were confined to a small group of female friends within her religious community, and she did not seek age-appropriate intimate relationships. Following episodes of bullying during primary school and cyberbullying during high school, she showed early signs of psychic distress and a tendency to self-isolation. Lately, she developed body dysmorphic thoughts and aberrant eating behaviors, leading her to excessive weight loss and amenorrhea when she was seventeen. At the same time, she started experiencing intrusive thoughts of profane, vulgar, and sexual content, compelling ideas of guilt and rituals of prayer and self-scratching. She therefore consulted a psychiatrist who started her on medication with sertraline, up to a dosage of 100 mg/day as first-line therapy in the initial hypothesis of an obsessive–compulsive presentation. Given the persistence of such symptoms and the emergence of subtle auditory perception abnormalities (i.e., hearing her parents calling her name, hearing judgmental voices), augmentation with risperidone was attempted up to a dosage of 3 mg/day. However, risperidone was discontinued early due to the occurrence of hyperprolactinemia and replaced by olanzapine, at a dosage of 2.5 mg/day.

### 2.2. Psychiatric Presentation, Diagnosis, and Clinical Course

By the time of her first outpatient visit at the Unit of Psychiatry, the patient was 18 years old. She was well kept, fully cooperative, and able to provide detailed medical history information. Her mood was euthymic. No signs of anxiety were observed. No hallucinatory behavior was observed. The patient showed a ruminative process of thinking and the thought content revealed worries about her own body image and weight control. She also reported recent episodes of binge eating and subsequent verbalization of suicidal intent, showing poor insight and flattened affect. Her sleep/wake rhythm was described as normal. She did not show any neurological signs or deficits. She did not appear grossly impaired on a cognitive basis, with both recent and remote memory preserved. Orientation in space, time, and person was intact. A thorough psychological assessment was then carried out using the Eating Disorder Inventory (EDI)-3, the Body Uneasiness Test (BUT), the Symptom Checklist (SCL)-90, the Binge Eating Scale (BES), the Minnesota Multiphasic Personality Inventory (MMPI)-2, the Structured Clinical Interview for Diagnostic and Statistical Manual of Mental Disorders, -Fifth Edition, -Clinician Version (SCID-5-CV), and the SCID-5, -Personality Disorders (SCID-5-PD), converging towards the diagnosis of other specified feeding or eating disorder (OSFED) and prior major depressive episode, according to the Diagnostic and Statistical Manual of Mental Disorders, -Fifth Edition (DSM-5): in particular, characteristics of drive for thinness, weight phobia, compulsive control of body image, tendency to perfectionism, personal insecurity, somatization, and emotional dysregulation emerged. On the contrary, the presence of a personality disorder was ruled out. Ongoing pharmacotherapy with sertraline was maintained at first and olanzapine was progressively titrated to 7.5 mg/day and then tapered off and discontinued due to excessive sedation. Psychotherapy intervention and monitoring of the eating disorder were initiated and continued for about one year. At a follow-up visit planned one year after her first evaluation, a good control over aberrant eating behaviors was reported, whereas egodystonic blasphemous thoughts persisted, often followed by the urge to perform specific movements or acts (e.g., running to exhaustion, screaming, beating the wall, beating her chest), of which the patient felt ashamed. Also, motor and vocal tics including echopraxia (i.e., repeatedly writing down sentences pronounced by others), echolalia (i.e., verbally repeating sentences pronounced by others), and tongue clicking emerged at that time. During the next 3 years, the clinical picture was monitored on an almost monthly basis. Despite good compensation of affective symptoms, the eating disorder, and early perceptual distortions, monitoring indicated that the intrusive thoughts, repetitive behaviors, and tics kept fluctuating. A brain MRI scan taken when she was 19 years old revealed consistent findings with the previous one, confirming the presence of DWV. A neuropsychological assessment was performed using the Wechsler Adult Intelligence Scale, -Fourth Edition (WAIS-IV). The patient’s full-scale intelligence quotient (FSIQ) was in the average range as per the normative data (FSIQ = 95), with “Verbal Comprehension” hard to interpret due to high internal discrepancy, “Perceptual Reasoning” and “Verbal Memory” in the average range, and “Processing Speed” in the very low average range. Further assessment using the Raven’s Standard Progressive Matrices (SPM) confirmed an average intelligence. Moderate deficits in executive function and spatial cognition were highlighted with the Cerebellar Cognitive Affective syndrome (CCAS) scale. The reported level of personal autonomy was investigated through the World Health Organization Disability Assessment Schedule (WHODAS) 2.0, which revealed mildly impaired general functioning. Individual difficulties were reported in the domains of Comprehension and Communication, Activities of Daily Living, and Social Interaction. A differential diagnosis with full-blown psychotic disorders was considered. However, the patient easily dismissed the puzzling auditory experiences occurring prior to her presentation to the Unit, which were recognized as intrusive thoughts that were just her imagination. Also, repetitive behaviors were perceived as egodystonic and not enacted in response to the delusional content of hallucinations. No other potential psychotic symptoms were detected for the entire duration of the healthcare path. Consequently, a final diagnosis of obsessive–compulsive disorder (OCD) associated with persistent motor and vocal tic disorder was made, according to the DSM-5. Throughout the years, several attempts were made to adjust pharmacotherapy, never resulting in complete remission of symptoms. First, sertraline as monotherapy was titrated to 200 mg/day, but obsessive–compulsive symptoms and tics did not subside. Aripiprazole at a dosage of 10 mg/day was then added to sertraline, with beneficial effects over intrusive thoughts and tics, but with early occurrence of constipation and a sudden drop in white blood cell count and platelet count, so it was reduced to a dosage of 5 mg/day with relapsing symptoms. Adjunctive therapy with haloperidol up to a dosage of 4 mg/day was also beneficial on intrusive thoughts but led to the occurrence of increasing anxiety and daytime sedation, so it was suspended concurrently with aripiprazole. Quetiapine extended release (ER; 50 mg/day) and subsequent lurasidone (74 mg/day) add-on attempts were not effective and quickly switched to risperidone (up to 3 mg/day), which the patient asked to stop after about one year due to excessive weight gain, although a good control over symptoms was observed. Ongoing sertraline was then cross-titrated with clomipramine (150 mg/day) as a third-line treatment for OCD, leading to a rapid improvement in obsessive thinking, but suspended shortly after due to the occurrence of iatrogenic acute hepatitis. The patient was then started on brexpiprazole (up to 4 mg/day, then lowered to the present dosage of 2 mg/day), which has been continued for over one year and a half at the time of writing.

Following the initiation of therapy, the patient has reported substantial subjective well-being and stable mood. Obsessive thoughts have persisted, but these did not affect general functioning. Compulsions and tics have ameliorated. No adverse effects have been noticed following the initiation of therapy. The patient was able to complete a bachelor’s degree course at university with good grades and reported improved social drive.

## 3. Systematic Review

### 3.1. Methods

The review followed the Preferred Reporting Items for Systematic Reviews and Meta-Analyses (PRISMA) 2020 guidelines [14]. A literature search was performed using the electronic databases PubMed, EMBASE, and Cochrane Library, using a combination of the following MESH terms describing and/or concerning DWC abnormalities (“Dandy–Walker”, “Blake pouch cyst”, “mega cisterna magna”, “Chudley–Mccullough syndrome”) and psychiatric symptoms or neurodevelopmental/neuropsychological conditions (“mental disorder”, “psychiatric disorder”, “psychosis”, “psychotic”, “behavioral disorder”, “obsessive–compulsive disorder”, “depression”, “depressive disorder”, “schizophrenia”, schizophrenic disorder”, “mania”, “manic disorder”, “bipolar disorder”, “tic”, “Tourette”, “autism”, “autistic disorder”, “ADHD”, “neurodevelopmental disorder”, “intellectual disability”, “cognitive impairment”, “neurocognitive impairment”, “dementia”, “learning retardation”, “motor coordination retardation”, “learning delay”, “motor coordination retardation delay”). The search was conducted on 10 January 2023. The exhaustive search string is available at: https://www.crd.york.ac.uk/PROSPEROFILES/416704_STRATEGY_20230414.pdf (accessed on 28 March 2024).

Studies were eligible for inclusion in this review if they investigated psychiatric symptoms in adult patients (≥18 years of age) with DWC abnormalities. We included only original papers with a case report or case series design published in English in peer-reviewed journals. No predefined time window for the study se arch was adopted, to be as inclusive as possible. By using a three-step screening approach, articles were screened through title, abstract, and full-text reading, if needed. Studies were excluded if they (i) had a design other than case report or case series or did not report original data; (ii) reported on children and adolescents; (iii) did not report on psychiatric symptoms or neurodevelopmental/neuropsychological conditions in patients with DWC abnormalities presenting to psychiatric in-patient or out-patient settings; or (iv) did not provide a clear description of either psychiatric, behavioral, neurodevelopmental, or neuropsychological characteristics of the subject(s).

The screening and data extraction were done by using an web-based systematic review management software (Covidence systematic review software, Veritas Health Innovation, Melbourne, Australia). Publication data screening and extraction were performed following a two-step selection process (conventional double-screening) conducted by two reviewers (R.B., A.C., A.N., and C.C.) at a time independently of each other. Further research evidence, gathered outside of the search or identified through manual search of the reference section of the included articles, was reported if considered appropriate by researchers. By applying a flexible approach, other articles that were deemed to cover prominent related topics were also searched by accessing grey literature and/or screening the reference lists of the eligible studies to provide a more comprehensive overview (See Figure 1). The following information was extracted from the included studies: study ID (including authors, year of publication, and country in which the study was conducted), study characteristics (including number of patients and patients’ sex, age, ethnicity, role, and social functioning), DW assessment (including neuroimaging description and genetics evaluation, where available), psychiatric and neurodevelopmental assessment (actual diagnosis and assessment tools, clinical setting), and psychotropic medication (both at admission and at discharge, including dosage, reported adverse events, and outcome).

Risk of bias (quality) assessment was performed by two reviewers (R.B. and C.C.) independently of each other, according to a set of criteria suggested by the Johanna Briggs Institute [15]. JBI is a globally recognized international collaboration specialized in evidence synthesis [16]. Over the past three decades, it has developed a comprehensive set of tools spanning various designs, structures, and applications, including case reports and case series, among others [17,18,19]. The JBI tool for case reports consists of an eight-item scale exploring the patient’s demographic characteristics, medical history, clinical details, details of laboratory work-up, intervention(s) or treatment procedure(s) given, follow-up clinical condition, adverse events, and the key message from the case reports. The JBI critical appraisal consists of four evaluation items: “Yes”, “No”, “Unclear”, and “Not Applicable”. Affirmative items are summarized as a score from 0 to 8. Case reports with a score lower than 4 were classified as having a low quality, those with a score between 4 and 6 as having a medium quality, and those with a score higher than 6 were considered high quality. In the rare instances of discrepant screening, data extraction, or quality assessment, a consensus was sought through discussion with a third senior clinical researcher (M.C.). The full study protocol (PROSPERO 2023 CRD42023416704) is available at: https://www.crd.york.ac.uk/prospero/display_record.php?ID=CRD42023416704 (accessed on 28 March 2024).

### 3.2. Results

#### 3.2.1. Study Selection

**Figure 2:**
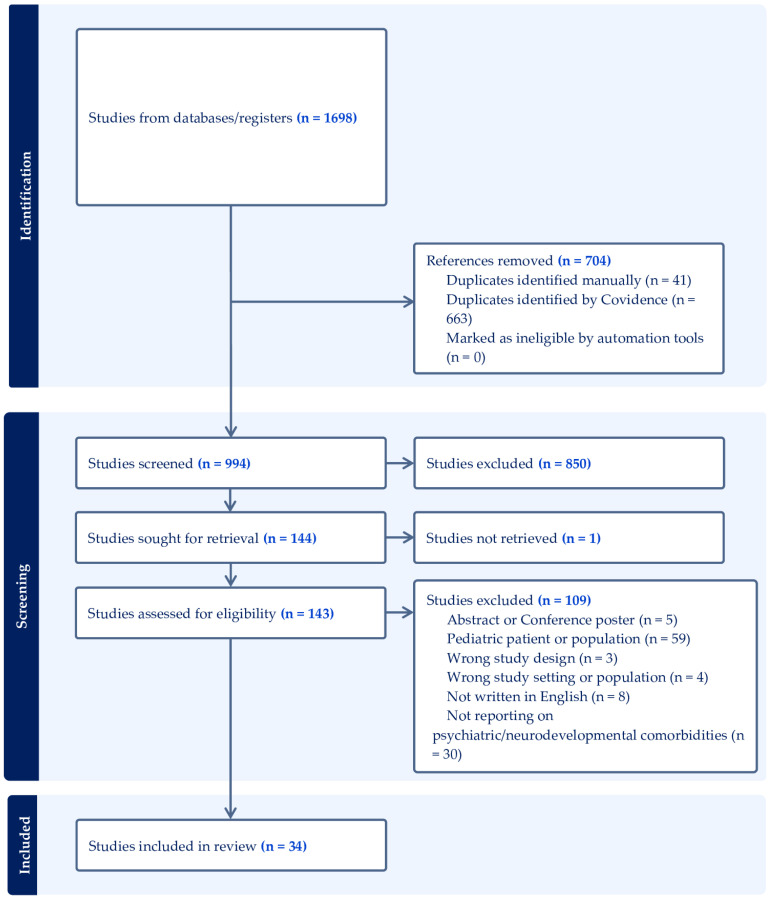
PRISMA flow-chart of search strategy for systematic review.

Altogether, 1698 articles were imported for screening. After removing duplicates, 994 papers were retrieved. By using a three-step screening approach, titles, abstracts, or full texts of all records were screened against the inclusion and exclusion criteria (Figure 2). The systematic review comprised thirty-four articles, consisting of thirty-two case reports and two case series. A brief synthesis of the main results is presented below and summarized in Table 1.

All included studies underwent critical appraisal. A single study was classified as having lower methodological rigor [20], as assessed through the JBI critical appraisal tool [15]. Nevertheless, no articles were excluded owing to a low appraisal score. The detailed risk of bias and quality assessment with the corresponding questions for each item and list of case reports is shown in Table 2.

**Table 1:** Summary characteristics of included case reports.

Study ID	Country	Age, Gender	Neuroimaging Description	Neurodevelopmental Comorbidity	Psychiatric Comorbidity	Clinical Setting	Psychotropic Medication at Presentation	Psychotropic Medication at Discharge	Outcome
Aune and Bugge, 2014 [21]	NO	22, M	DWV (CT, MRI)	Cognitive impairment (WAIS-III, WISC-Revised) Learning disorder (dyslexia) Attention deficit	Schizophrenia (CE, BPRS, MINI)	Inpatient	Risperidone (1 mg/day, oral)	Risperidone (2 mg/day, oral)	Complete remission
Bakhla et al., 2010 [22]	IN	24, M	DWM (CT)	-	Bipolar I disorder (CE)	Inpatient	Lithium (1350 mg/day, oral) Olanzapine (20 mg/day, oral)	Sodium valproate (1500 mg/day, oral) Carbamazepine (400 mg/day, oral) Olanzapine (15 mg/day, oral)	Complete remission
Balcioglu et al., 2018 [23]	TR	36, M	MCM (MRI)	-	Psychosis NOS (CGI, MMSE, PANSS, Rorschach test)	Inpatient Compulsory	-	Risperidone (3 mg/day, oral) Quetiapine (300 mg/day, oral)	Complete remission
Batmaz et al., 2017 [24]	TR	27, M	DWM (MRI)	Psychomotor delay Learning disorder Borderline cognitive functioning (WISC-Revised, WAIS)	Bipolar I disorder (YMRS, CE)	Inpatient Compulsory	Aripiprazole (30 mg/day, oral) Quetiapine (400 mg/day, oral)	Aripiprazole (30 mg/day, oral) Quetiapine (200 mg/day, oral) Sodium valproate (1000 mg/day, oral) Biperiden (4 mg/day, oral) Paliperidone palmitate (100 mg/month, IM)	Complete remission
Blaettner et al., 2015 [25]	AT	19, M	DWV (MRI)	-	Delusional disorder (CE, SCID)	Inpatient	-	Ziprasidone (120 mg/day, oral) Trazodone (150 mg/day, oral)	Complete remission
Bout et al., 2021 [26]	MA	20, M	DWV (MRI)	-	Schizophrenia (PANSS, CDSS) Depression	Inpatient	Risperidone (2 mg/day, oral)	Fluoxetine (20 mg/day, oral) Quetiapine (600 mg/day, oral)	Partial remission
Bozkurt Zincir et al., 2014 [27]	TR	30, F	DWV (MRI)	Borderline cognitive functioning (WAIS-Revised)	Schizophrenia (CE, PANSS)	Inpatient	-	Risperidone (50 mg, depot injection) Risperidone (6 mg/day, oral) Biperiden (4 mg/day, oral) Lorazepam (2.5 mg/day, oral)	Partial remission
Buonaguro et al., 2014 [28]	IT	29, F	DWM (MRI)	-	Psychosis NOS (CE, PANSS, SCID, MMSE, MMPI, BACS, WAIS-R)	Inpatient	Sodium valproate (750 mg/day, oral) Haloperidol (4 mg/day, oral) Biperiden (4 mg/day, oral)	-	-
Can et al., 2014 [29]	TR	32, M	DWV (MRI)	Attention deficit, working memory deficit, learning difficulties (BGVMSS, BVMMS, WMS)	Bipolar I disorder (CE, YMRS, MMPI)	Outpatient	Lithium (dose not given, oral) Sodium valproate (dose not given, oral) Risperidone (dose not given, oral) Biperiden (dose not given, oral)	Sodium valproate (1250 mg/day, oral) Quetiapine (500 mg/day, oral)	Partial remission
Dawra et al., 2017 [30]	IN	18, M	DWV (MRI)	Cognitive impairment	Psychosis NOS (CE)	Inpatient	Sodium phenytoin (100 mg, 3 times/day) Carbamezapine (200 mg, 3 times/day)	Risperidone (2 mg/day, oral); Trihexyphenidyl (2 mg/day, oral)	-
El Tahir et al., 2022 [31]	QA	18, M	DWM (MRI)	Mild intellectual disability (WISC-III)	Intermittent explosive disorder (CE)	-	Topiramate (dose not given, oral)	Sodium valproate (dose not given, oral) Risperidone (dose not given, oral)	Complete remission
Ferentinos et al., 2007 [32]	GR	21, F	MCM (CT, MRI)	Borderline cognitive functioning (ST, WCST, RCF)	Psychosis NOS (CE, PANSS, SOFAS)	Outpatient	Amisulpride (1200 mg/day, oral)	Amisulpride (400 mg/day, oral) Galantamine (8 mg/day, oral)	Complete remission
Gama Marques, 2019 [33]	PT	54, F	DWV (CT)	Moderate intellectual disability	Delusional disorder (CE)	Outpatient	Haloperidol (150 mg/monthly, depot injection) Biperiden (4 mg/day, oral) Venlafaxine (150 mg/day, oral)	Haloperidol (100 mg/monthly, depot injection) Biperiden (4 mg/day, oral) Venlafaxine (150 mg/day, oral)	Complete remission
Gan et al., 2012 [34]	CN	Patient 1: 45, M Patient 2: 20, M	Patient 1: MCM (MRI) Patient 2: PFAC (MRI)	Patient 1: Mild intellectual disability (CE) Patient 2: Mild intellectual disability (CE, WAIS-Revised, WMS)	Patient 1: Schizophrenia (CE, BPRS, CGI) Patient 2: Bipolar II disorder (CE, HRSD, BRMRS)	Patient 1: Inpatient Compulsory Patient 2: Inpatient Compulsory	Patient 1: Clozapine (200 mg/day, oral) Patient 2: Duloxetine (90 mg/day, oral) Olanzapine (15 mg/die, oral); Clonazepam (2 mg/day, oral)	Patient 1: (a) First discharge: Clozapine (200 mg/day, oral); (b) Second discharge: Risperidone (7 mg/day, oral) Patient 2: (a) First discharge: Duloxetine (90 mg/day, oral) Olanzapine (15 mg/day, oral) Clonazepam (2 mg/day, oral); (b) Second discharge: Olanzapine (20 mg/day, oral) Sodium valproate (1.2 g/day, oral) Clonazepam (2 mg/day, oral)	Patient 1: Complete remission Patient 2: Complete remission
Graf et al., 2013 [35]	DE	26, M	DWV (MRI)	Psychomotor delay, Language development delay, Learning disorder, Attention-deficit disorder	Intermittent explosive disorder (CE)	Outpatient	-	Quetiapine (dose not given, oral)	-
Iancu et al., 1996 [36]	IL	19, F	DWM (CT, MRI)	-	Psychogenic nonepileptic seizures (CE, EEG)	Inpatient	-	-	Complete remission
Isidro-Garcia et al., 2017 [37]	ES	34, F	DWV (CT, MRI)	-	Schizophrenia (CE, PANSS)	Outpatient	Olanzapine (up to 20 mg/day, oral) Haloperidol (up to 10 mg/day, oral) Paliperidone (up to 12 mg/day, oral)	Clozapine (up to 600 mg/day, oral) Haloperidol (5 mg/day, oral)	Partial remission
Kani et al., 2015 [38]	TR	57, F	MCM (MRI)	Borderline cognitive functioning	Schizophrenia (CE, PANSS) Obsessive–compulsive disorder (CE, Y–BOCS)	Outpatient	Risperidone (4 mg/day, oral) Quetiapine (300 mg/day, oral) Clomipramine (225 mg/day, oral)	Risperidone (4 mg/day, oral) Quetiapine (300 mg/day, oral) Fluvoxamine (300 mg/day, oral) Clonazepam (1 mg/day, oral)	Complete remission
Kim et al., 2013 [39]	KR	33, M	DWV (MRI)	-	Depression (CE, HRSD, BDI) Impulsive behavior (CE)	Inpatient Compulsory	-	Mirtazapine (45 mg/day, oral) Sodium valproate (1500 mg/day, oral) Quetiapine (800 mg/day, oral)	Partial remission
Kumar et al., 2011 [40]	IN	37, F	MCM (MRI)	-	Schizophrenia (CE)	Inpatient	Lorazepam (2 mg three times/day, intramuscular)	-	Relapsing symptoms after discharge
Li et al., 2008 [20]	CN	40, M	DWM (MRI)	-	Bipolar II disorder (CE)	Inpatient	Venlafaxine (dose not given, oral)	Lithium (dose not given, oral) Quetiapine (dose not given, oral) Sertraline (dose not given, oral)	-
Mauritz et al., 2014 [41]	NL	47, F	DWM (-)	-	Posttraumatic Stress Disorder (CE, GAF, DTS, SIDES, DES, PANSS)	Inpatient Outpatient	Quetiapine (600 mg/day, oral)	Quetiapine (600 mg/day, oral) Citalopram (40 mg/day, oral)	Partial remission
Ozcan and Ulkevan, 2015 [42]	TR	34, M	DWV (CT)	-	Bipolar I disorder (CE)	Inpatient	Olanzapine (10 mg/day, oral)	Olanzapine (15 mg/day, oral) Lithium (900 mg/day, oral) Lorazepam (2.5 mg day, oral)	Complete remission
Pandurangi et al., 2014 [43]	IN	Patient1: 26, M Patient 2: 20, M	Patient 1: MCM (CT) Patient 2: MCM (MRI)	Patient 1: - Patient 2: -	Patient 1: Bipolar I disorder (CE, YMRS) Patient 2: Schizophrenia (CE, BFCRS)	Patient 1: Outpatient Patient 2: Inpatient	Patient 1: - Patient 2: -	Patient 1: Lithium (800 mg/day) Risperidone (4 mg/day) Patient 2: Risperidone (3 mg/day)	Patient 1: Complete remission; Patient 2: -
Papazisis et al., 2007 [44]	GR	20, M	DWV (CT, MRI)	Mild intellectual disability (WISC-Revised)	Schizophrenia (CE), Obsessive–compulsive disorder (CE, Y–BOCS)	Inpatient	-	Antipsychotic (dose not given, route not given) Antidepressant (dose not given, route not given)	Relapsing symptoms after discharge
Porras Segovia et al., 2021 [45]	ES	32, M	DWV (MRI)	Mild intellectual disability (WAIS-III)	Schizophrenia (CE, PANSS)	Inpatient	-	Olanzapine (10 mg/day, oral)	Partial remission
Pradhan et al., 1998 [46]	IN	33, M	DWM (CT)	Borderline cognitive functioning (LNNB)	Psychosis NOS (CE)	Inpatient	Diphenylhydantoin (300 mg/day, oral) Phenobarbiton (120 mg/day, oral)	Carbamazepine (800 mg/day, oral)	Complete remission
Sidana et al., 2013 [47]	IN	20, M	DWV (CT)	-	Schizophrenia (CE)	-	Olanzapine (10 mg/day, oral)	Aripiprazole (2.5 mg/day, oral)	-
Sinha et al., 2017 [48]	IN	25, M	DWV (MRI)	Motor development delay Borderline cognitive functioning (WAPIS)	Schizophrenia (CE)	Inpatient	Olanzapine (15 mg/day, oral)	Olanzapine (15 mg/day, oral)	Partial remission
Trehout et al., 2018 [49]	FR	24, M	DWM (MRI)	-	Schizophrenia (CE, CAARMS, MINI, PANSS, BPRS)	Inpatient	Olanzapine (5 mg/day, oral)	Clozapine (600 mg/day, oral) Loxapine (dose not given, oral)	Partial remission
Turan et al., 2010 [50]	TR	23, M	MCM (MRI)	-	Bipolar I disorder (CE, YMRS)	Inpatient	Olanzapine (20 mg/day, oral)	Quetiapine (1000 mg/day, oral) Sodium valproate (1500 mg/day, oral)	Complete remission
Turner et al., 2001 [51]	UK	18, F	DWV (CT, MRI)	-	Schizophrenia (CE, WAIS)	Inpatient Compulsory	Fluoxetine (dose not given, oral)	-	Complete remission
Williams et al., 2016 [52]	USA	20, F	DWV (CT, MRI)	Autism spectrum disorder	Schizoaffective disorder (CE)	Inpatient	Haloperidol (7 mg/day, oral)	Olanzapine (20 mg/day, oral)	Relapsing symptoms after discharge
Yazici et al., 2022 [53]	TR	28, F	MCM (MRI)	-	Bipolar I disorder (RCF, MMPI)	Inpatient	Quetiapine (100 mg/day, oral) Haloperidol (10 mg/day, oral) Biperiden (2.5 mg/day, injection)	Quetiapine (100 mg/day, oral) Olanzapine (20 mg/day, oral) Lithium (900 mg/day, oral)	Complete remission

AT: Austria; BACS: Brief Assessment of Cognition in Schizophrenia; BDI: Beck Depression Inventory; BFCRS: Bush Francis Catatonia Rating Scale; BGVMSS: Bender Gestalt Visual Motor Sensation Scale; BPRS: Brief Psychiatric Rating Scale; BRMRS: Bech–Rafaelsen Mania Rating Scale; BVMMS: Benton Visual Motor Memory Scale; CAARMS: Comprehensive Assessment of at Risk Mental States; CDSS: Calgary Depression Scale for Schizophrenia; CE: Clinical Examination; CGI: Clinical Global Impression; CN: China; CT: Computed Tomography; DE: Germany; DES: Dissociative Experiences Scale; DTS: Davidson Trauma Scale; DWM: Dandy–Walker Malformation; DWV: Dandy–Walker Variant; EEG: electroencephalogram; ES: Spain; F: Female; FR: France; GAF: Global Assessment of Functioning; GR: Greece; HRSD: Hamilton Rating Scale for Depression; HWSISC-III: Hamburg–Wechsler Scale Intelligence Scale for Children III; IL: Israel; IN: India; IT: Italy; KR: Republic of Korea; LNNB: Luria–Nebraska Neuropsychological Battery; M: Male, MA: Morocco; MCM: Mega Cisterna Magna; MINI: Mini International Neuropsychiatric Interview; MMPI: Minnesota Multiphasic Personality Inventory; MMSE: Mini-Mental State Examination; MRI: Magnetic Resonance Imaging; NL: The Netherlands; NO: Norway; NOS: Not Otherwise Specified; PANSS: Positive And Negative Symptoms Scale; PFAC: Posterior Fossa Arachnoyd Cyst; PT: Portugal; QA: Qatar; RCF: Rey Complex Figure; SCID: Structured Clinical Interview for DSM; SIDES: Structured Interview for Disorders of Extreme Stress; SOFAS: Social and Occupational Functioning Assessment Scale; ST: Stroop test; TR: Turkey; UK: United Kingdom; USA: United States of America; WAIS: Wechsler Adult Intelligence Scale; WAPIS: Wechsler Adult Performance Intelligence Scale; WCST: Wisconsin Card Sorting Test; WISC: Wechsler Intelligence Scale for Children; WMS: Wechsler memory scale; Y–BOCS: Yale–Brown Obsessive Compulsive Scale; YMRS: Young Mania Rating Scale.

#### 3.2.2. Characteristics of Studies Included

Studies were published between 1996 and 2022 and were conducted across 18 different countries, with 44.4% of them being performed in Turkey, 38.9% in India, and 16.7% in other countries. Thirty-six patients were involved, twenty-four of which were male (66.7%). The mean age was 28.6 ± 10.2 years old.

Overall, twenty-two patients (61.1%) underwent only an MRI scan [20,23,24,25,26,27,28,29,30,31,34,35,38,39,40,43,45,48,49,50,53], six patients (16.7%) underwent only a computed tomography (CT) scan [22,33,42,43,46,47], seven patients (19.4%) underwent both an MRI and a CT scan [21,32,36,37,44,51,52], and in a single case (2.8%), no neuroimaging investigations were reported [41]. Neuroimaging procedures revealed DWV in seventeen cases (47.2%) [21,25,26,27,29,30,33,35,37,39,42,44,45,47,48,51,52], isolated MCM in nine cases (25%) [23,32,34,38,40,43,50,53], DWM in nine cases (25%) [20,22,24,28,31,36,41,46,49], and isolated PFAC in one case (2.8%) [34]. Cerebellar lesions were reported in twenty-two cases (61.1%) [20,21,22,24,26,27,28,29,30,31,33,35,36,37,39,44,45,47,48,49,51,52], including vermis [20,21,22,24,26,27,28,29,30,31,33,35,36,37,39,44,45,47,48,49,51,52] and/or hemisphere [31,36,49,51] hypoplasia, dysplasia, or agenesis. Twenty patients (55.6%) presented ventricular system abnormalities and/or dilatations [20,21,22,24,27,28,29,30,35,36,39,41,43,44,45,46,47,48,49]. Enlarged posterior fossa CSF space was described in sixteen cases (44.4%) [22,23,24,26,28,29,32,33,34,38,39,40,46,50,52,53], including enlarged cisterna magna [23,26,28,29,32,33,34,38,39,40,50,53] and posterior fossa cysts [24,34,46,52]. Interestingly, most patients (83.3%) were incidentally diagnosed with DWC abnormalities only after presenting with acute psychiatric symptoms, prompting subsequent neuroimaging investigations [21,22,23,25,26,27,28,29,32,33,34,37,38,39,40,43,44,45,46,47,48,49,50,51,52,53].

Only two studies reported on genetic evaluations, describing a normal karyotype [28,32].

#### 3.2.3. Neurodevelopmental/Neuropsychological Comorbidities and Psychiatric/Behavioral Phenotypes

Cognitive impairment was detected in 14 cases (38.9%) [21,24,27,30,31,32,33,34,38,44,45,46,48], ranging from borderline cognitive functioning [24,27,32,38,46,48] to mild [31,34,44,45] or moderate [33] intellectual disability. Similar but not overlapping methodologies were used in terms of assessment tools for cognitive functioning, including the WAIS [21,24,27,34,45], the Wechsler Intelligence Scale for Children (WISC) [21,24,31,44], the Wechsler Memory Scale (WMS) [29,34], the Wechsler Adult Performance Intelligence Scale (WAPIS) [48], the Luria–Nebraska Neuropsychological Battery (LNNB) [46], the Stroop test (ST) [32], the Wisconsin Card Sorting test (WCST) [32], the Rey Complex Figure (RCF) [32], the Bender Gestalt Visual Motor Sensation Scale (BGVMSS) [29], and the Benton Visual Motor Memory Scale (BVMMS) [29]. Other comorbid neurodevelopmental/neuropsychological conditions encompassed learning disorders (8.3%) [21,24,35], attention-deficit disorder and sub-clinic attention deficiency (8.3%) [21,29,35], and global psychomotor delay (5.6%) [24,35]. In addition, single cases of comorbid ASD [52], delayed motor development [48], delayed language development [35], and subtle learning difficulties [29] were reported.

Twenty-two patients (61.1%) presented with comorbid psychosis, including schizophrenia [21,26,27,34,37,38,40,43,44,45,47,48,49,51], psychotic disorder not otherwise specified (NOS) [23,28,30,32,46], delusional disorder [25,33], and schizoaffective disorder [52]. Similar but not overlapping methodologies were used in terms of assessment tools investigating psychotic symptoms [the Positive and Negative Symptoms Scale (PANSS) [23,26,27,28,32,37,38,45,49], the Brief Psychiatric Rating Scale (BPRS) [21,34,49], the Comprehensive Assessment of At-Risk Mental States (CAARMS) [49], the Bush Francis Catatonia Rating Scale (BFCRS) [43], the Rorschach test [23]], psychosis-related depressive symptoms [the Calgary Depression Scale for Schizophrenia (CDSS) [26]], general psychopathology [the Mini International Neuropsychiatric Interview (MINI) [21,49], the SCID [25,28], the MMPI [28], cognition [the Brief Assessment of Cognition in Schizophrenia (BACS) [28], the Mini-Mental State Examination (MMSE) [23,28], the WAIS [28,51], and global functioning [the Social and Occupational Functioning Assessment Scale (SOFAS) [32], the Clinical Global Impression (CGI) [23,34]. In few cases, a psychotic disorder was diagnosed solely according to clinical examination [30,33,40,44,46,47,48,52].

Affective disorders were detected among eleven patients (30.6%). Of these cases, nine presented with bipolar disorder [20,22,24,29,34,42,43,50,53] and two presented with depression [26,39]. Mood disorders and symptoms were assessed using the Young Mania Rating Scale (YMRS) [24,29,43,50], the Hamilton Rating Scale for Depression (HRSD) [34,39], the Beck Depression Inventory (BDI) [39], the Bech–Rafaelsen Mania Rating Scale (BRMRS) [34], the CDSS [26], and the MMPI [29,53]. All other cases of mood disorders were diagnosed only based on clinical examination [20,22,42]. OCD was diagnosed in two cases (5.6%), using the Yale–Brown Obsessive Compulsive Scale (Y–BOCS) [38,44]. Other comorbid psychiatric/behavioral conditions comprised intermittent explosive disorder [31,35], impulsivity [39], psychogenic nonepileptic seizures [36], and posttraumatic stress disorder [41].

#### 3.2.4. Use of Psychopharmacological Medications and Clinical Course

Most patients were initially admitted to an inpatient setting (n = 27; 75%), due to acute psychiatric manifestations [20,21,22,23,24,25,26,27,28,30,34,36,39,40,41,42,43,44,45,46,48,49,50,51,52,53], in some cases requiring compulsory admission [23,24,34,39,51]. Conversely [31,32,33], only eight patients were exclusively assessed on an outpatient basis [29,32,33,35,37,38,41,43].

At first presentation, 10 patients (27,8%) were not taking any medications [23,25,27,35,36,39,43,44,45], 15 patients (41.7%) were on monotherapy [20,21,26,31,32,34,40,41,42,47,48,49,50,51,52], and 11 patients (30.5%) were on polytherapy [22,24,28,29,30,33,34,37,38,46,53]. The most prescribed drugs at presentation were second- [21,22,24,26,29,32,34,37,38,41,42,47,48,49,50,53] and first-generation [28,33,37,52,53] antipsychotics, followed by mood stabilizing agents [22,28,29,30,31,46], antidepressants [20,33,34,38,51], and anxiolytics and hypnotic/sedatives [34,40,46]. At discharge, only four patients (11.1%) were not being prescribed any medications (with a 60% decrease compared to presentation) [28,36,40,51], nine patients (25%) were on monotherapy (with a 40% decrease compared to presentation) [21,34,35,43,45,46,47,48,52], and 23 patients (63.9%) were on polytherapy (with a 109% increase compared to presentation) [20,22,23,24,25,26,27,29,30,31,32,34,37,38,39,41,42,43,44,49,50,53]. Among patients who were not taking any medications, one underwent electroconvulsive therapy [40]. Consistent with presentation, the most prescribed drugs among treated patients were second- [21,23,24,25,26,27,29,30,31,32,34,35,37,38,41,42,43,45,47,48,49,50,52,53] and first-generation antipsychotics [33,37,49], followed by mood stabilizing agents (with a 100% increase compared to presentation) [20,22,24,29,31,34,39,42,43,46,50,53], antidepressants (with an 80% increase compared to presentation) [20,25,26,33,34,38,39,41,44], and anxiolytics and hypnotic/sedatives [27,34,38,42]. Outcome information was available for 30 patients. Among them, eighteen patients (60%) were in complete remission [21,22,23,24,25,31,32,33,34,36,38,42,43,46,50,51,53], nine patients (30%) were in partial remission [26,27,29,37,39,41,45,48,49], and three patients (10%) relapsed after discharge [40,44,52].

## 4. Discussion

Here, we illustrate the case of a young woman with DWV displaying a severe and complex neuropsychiatric presentation. Consistent with the previous research in the field, early language development difficulties influenced the patient’s poor social adjustment during schoolyears, possibly leading to episodes of peer victimization and a tendency to isolation [54]. Together with social difficulties, her siblings’ disability may have had an impact on the development of much clearer psychic distress during the transition from late adolescence to young adulthood [55,56,57], encompassing depressive symptoms, aberrant eating behaviors, obsessive–compulsive symptoms, and tics. To this extent, the previous literature has already documented instances of both adults and children with DWC abnormalities presenting with concurrent depression or OCD [26,38,39,44,58]. On the contrary, DWC abnormalities and comorbid persistent tic disorder have never been described so far. According to recent studies, alterations in cerebellar microstructural white matter fibers may underlie obsessive–compulsive symptoms and tic syndromes [59,60], thus warranting the need to monitor their occurrence among patients with posterior fossa lesions. Also, whereas the single case of a syndromic patient with a feeding disorder was previously reported [61], to the best of our knowledge this is the first case detailing an adult patient with DWV and comorbid OSFED. Previous studies have already underscored how altered cerebellar gray matter volume may fuel the persistence of anorexia nervosa, supporting the maintenance of low body weight and starvation, but current evidence on the role of the cerebellum in the physiopathology of other eating disorders is still in its infancy [62].

During the observation period, the patient underwent several pharmacological treatments, all of which were discontinued due to transient or suboptimal symptom management, as well as the rapid occurrence of adverse events. Eventually, the patient was treated with brexpiprazole as monotherapy, with good tolerance and improved compulsive symptoms and tics, aligning with earlier, although scarce, evidence on the subject [63]. 

It is worth mentioning that the use of antipsychotics (both as add-on therapy and as monotherapy) has always been intended to reduce difficult-to-treat obsessive–compulsive symptoms and tics [64,65]. This approach is particularly required when selective serotonin reuptake inhibitors (SSRIs) and tricyclic antidepressants (TCAs) have to be discontinued because of insufficient symptom management or unpleasant side effects. Both instances might be explained by the conflicting evidence about the efficacy, safety, and tolerability of SSRIs and TCAs among individuals with neurodevelopmental disorders [66,67,68].

Afterwards, we systematically reappraised all case reports and case series exploring the psychiatric burden of adult patients with DWC abnormalities. Overall, the present review demonstrated that psychiatric phenotypes are more represented among young males with posterior fossa lesions. In many cases, DWC abnormalities are discovered incidentally through neuroimaging assessments following patients’ admission to psychiatric inpatient facilities during acute illness episodes. The most common clinical subtype is DWV, particularly entailing lesions of the cerebellar vermis. Psychosis and bipolar disorder stand as the most diagnosed psychiatric conditions, often preceded by concurrent neurodevelopmental susceptibilities, primarily characterized by varying degrees of cognitive impairment. Finally, as indicated by reported studies, most patients recover from their initial acute episodes, often requiring polypharmacological approaches.

Some important findings from our systematic review deserve to be highlighted. First, we found that 2/3 patients with DWC abnormalities and psychiatric symptoms were men, with psychotic and affective disorders being the most frequently diagnosed. In the general population, there are no gender differences in the incidence of psychosis, even though male patients tend to onset earlier in life compared to females [69]. On the other hand, affective disorders tend to be more common in female patients compared to males [70]. Considering that cerebellar lesions were the predominant abnormalities observed among the selected cases [20,21,22,24,26,27,28,29,30,31,33,35,36,37,39,44,45,47,48,49,51,52], and acknowledging the potential influence of disrupted cortico-cerebellar connectivity and reduced cerebellar volumes on neuropsychiatric symptoms [7,8,10,11,13], as well as the documented higher gray matter volume in the cerebellum of females compared to males [71], the elevated prevalence of psychiatric symptoms in male patients compared to females suggests a gender-specific effect of DWC abnormalities on affective functions associated with the cerebellum.

Second, cerebellar anatomical abnormalities were the most prevalent among selected DWC patients [20,21,22,24,26,27,28,29,30,31,33,35,36,37,39,44,45,47,48,49,51,52], consistent with previous findings [5]. Not surprisingly, the majority of patients exhibited comorbid psychotic [21,23,25,26,27,28,30,32,33,34,37,38,40,43,44,45,46,47,48,49,51,52] or affective symptoms [20,22,24,26,29,34,39,42,43,50,53], for which clear neurobiological alterations involving the cerebellar vermis and hemispheres’ gray matter volumes, as well as reduced inter-connections with several brain areas regulating clinical presentations across the schizophrenia spectrum and mood disorders, seem to play a pivotal role [11,12,13] (Figure 1).

Third, it is intriguing that less than 50% of the cases provided information regarding neurodevelopmental vulnerabilities preceding the onset of psychiatric symptoms. Many patients exhibited discrete premorbid role functioning (e.g., high school graduated with/without special need support, university students, regular workers) [23,28,31,36,39,41,42,45,49,50,52,53] and social functioning (e.g., good personal care, having meaningful social contacts, living with partner and/or children) [23,37,40,41,46,50]. Additionally, approximately 30% of patients were not receiving any medications at the onset of their psychiatric symptoms [23,25,27,35,36,39,43,44,45] and over 80% had not already been diagnosed with DWC until adulthood [21,22,23,25,26,27,28,29,32,33,34,37,38,39,40,43,44,45,46,47,48,49,50,51,52,53]. These considerations may imply that the condition often progresses silently from childhood to late adolescence, only to manifest abruptly upon reaching young adulthood, possibly through the manifestation of psychic distress triggered by exposure to environmental factors [55,56,57].

Lastly, upon discharge, about 90% of patients received at least one medication, with the majority being prescribed psychopharmacological polypharmacy. Such an approach may reflect the need to address how DWC underlies multiple dysfunctional pathways between posterior fossa structures and brain areas (Figure 1). In comparison to their initial presentation, antipsychotics remained the most frequently prescribed, albeit with a slight decrease in the use of first-generation antipsychotics. However, the number of prescribed mood stabilizing agents doubled, and the number of prescribed antidepressants nearly doubled as well, reflecting common trends in medication management among adult patients with neurodevelopmental vulnerabilities [72].

Of course, the findings from this review should be seen considering some strengths and limitations. Despite confirming the frequency of long-term psychiatric sequelae in adulthood among patients with DWC abnormalities, the inherently biased nature of case reports and case series may have impacted the clinical data recollection and should not be overlooked. Indeed, although data emerging from the previous literature were systematically reappraised and a standardized quality of bias assessment tool was used, some case reports appeared to lack important information about the patients’ neurodevelopmental history, genetic profile, premorbid social and role adjustment, standardized psychometric assessments, after-treatment measures of outcome, and long-term course after discharge. Future high-quality CAse REport (CARE) guidelines-compliant studies will be needed to better investigate this subject.

Also, we provided the first evidence of comorbid tics and an eating disorder in an adult patient with DWV treated with brexpiprazole. It seems necessary to determine the frequency and nature of the above-mentioned symptoms in individuals with posterior fossa lesions, thus contributing to a better understanding of the underlying biobehavioral role of cerebellar abnormalities across psychiatric conditions.

## Figures and Tables

**Table 2:** Risk of bias and quality assessment.

Author/Question	Q1	Q2	Q3	Q4	Q5	Q6	Q7	Q8	Quality Appraisal
Aune and Bugge [21]	Y	N	Y	Y	Y	Y	U	Y	Medium
Bakhla et al. [22]	Y	N	Y	N	Y	Y	Y	Y	Medium
Balcioglu et al. [23]	Y	N	Y	Y	Y	N	N	Y	Medium
Batmaz et al. [24]	Y	Y	Y	Y	Y	N	Y	Y	High
Blaettner et al. [25]	Y	Y	Y	Y	Y	U	N	Y	Medium
Bout et al. [26]	Y	N	Y	Y	Y	Y	N	Y	Medium
Bozkurt Zincir et al. [27]	N	N	Y	Y	Y	Y	N	Y	Medium
Buonaguro et al. [28]	Y	Y	Y	Y	N	N	N	Y	Medium
Can et al. [29]	Y	N	Y	Y	Y	N	Y	Y	Medium
Dawra et al. [30]	Y	Y	Y	U	Y	Y	N	Y	Medium
El Tahir et al. [31]	Y	Y	Y	Y	N	Y	N	Y	Medium
Ferentinos et al. [32]	Y	N	Y	Y	Y	Y	Y	Y	High
Gama Marques [33]	Y	Y	Y	Y	Y	Y	N	Y	High
Gan et al. [34]	N	Y	Y	Y	Y	Y	N	Y	Medium
Graf et al. [35]	N	U	Y	Y	N	Y	N	Y	Medium
Iancu et al. [36]	Y	Y	Y	Y	N	Y	NA	Y	Medium
Isidro-Garcia et al. [37]	Y	Y	Y	Y	Y	U	N	Y	Medium
Kani et al. [38]	Y	Y	Y	Y	Y	Y	N	Y	High
Kim et al. [39]	Y	Y	Y	Y	Y	Y	N	Y	High
Kumar et al. [40]	Y	Y	Y	Y	Y	Y	N	Y	High
Li et al. [20]	N	N	Y	Y	N	N	N	Y	Low
Mauritz et al. [41]	Y	Y	Y	Y	Y	N	N	Y	Medium
Ozcan and Ulkevan [42]	Y	N	Y	N	Y	Y	N	Y	Medium
Pandurangi et al. [43]	Y	Y	Y	Y	Y	N	Y	Y	High
Papazisis et al. [44]	N	N	Y	Y	N	Y	N	Y	Medium
Porras Segovia et al. [45]	Y	Y	Y	Y	Y	Y	Y	Y	High
Pradhan et al. [46]	Y	Y	Y	Y	Y	Y	N	Y	High
Sidana et al. [47]	Y	N	Y	N	Y	N	Y	Y	Medium
Sinha et al. [48]	Y	Y	Y	Y	Y	Y	N	Y	High
Trehout et al. [49]	Y	Y	Y	Y	Y	Y	Y	Y	High
Turan et al. [50]	Y	N	Y	Y	Y	Y	Y	Y	High
Turner et al. [51]	Y	Y	Y	Y	N	Y	Y	Y	High
Williams et al. [52]	Y	Y	Y	U	Y	Y	Y	Y	High
Yazici et al. [53]	Y	Y	Y	Y	Y	N	N	Y	Medium

N: no; NA: not applicable; Qn: question; Y: yes; U: unclear.

## Data Availability

The original contributions presented in the study are included in the article; further inquiries can be directed to the corresponding author.
